# Challenges in the treatment of cystic fibrosis in the era of CFTR modulators

**DOI:** 10.36416/1806-3756/e20240107

**Published:** 2024-05-08

**Authors:** Caroline Jacoby Schmidt, Laura Silveira de Moura, Paulo de Tarso Roth Dalcin, Bruna Ziegler

**Affiliations:** 1. Programa de Pós-Graduação em Ciências Pneumológicas, Universidade Federal do Rio Grande do Sul - UFRGS - Porto Alegre (RS) Brasil.; 2. Serviço de Pneumologia, Hospital de Clínicas de Porto Alegre - HCPA - Porto Alegre (RS) Brasil.; 3. Serviço de Fisioterapia, Hospital de Clínicas de Porto Alegre - HCPA - Porto Alegre (RS) Brasil.

The paper entitled “Use of elexacaftor+tezacaftor+ivacaftor in individuals with cystic fibrosis and at least one F508del allele: a systematic review and meta-analysis,” published in the Brazilian Journal of Pulmonology, highlighted the effects of triple combination therapy targeting the cystic fibrosis transmembrane conductance regulator (CFTR) protein-elexacaftor+tezacaftor+ivacaftor (ETI). However, we believe that it is important to emphasize some challenges emerging regarding the maintenance of symptom treatment for the disease.

The maintenance of airway clearance techniques (ACTs) after the use of ETI is a challenge for professionals, families, and individuals with cystic fibrosis (CF). In addition to improving lung function and quality of life, as well as reducing the number of exacerbations, patients report a decrease in lung secretions with this therapy, leading to anxiety about the reduction of inhaled therapies (ITs) and ACTs.^1^ ^,^ ^2^

To date, no literature data supports the long-term reduction of ITs and ACTs. A randomized clinical trial with adults using ETI evaluated the suspension of ITs with hypertonic saline solution (SSH) or dornase alfa for six weeks. The authors observed non-inferiority in lung function with the suspension of one of the nebulization during the study follow-up. However, the group that suspended one of the ITs (SSH or dornase alfa) experienced a higher number of adverse effects when compared with the group maintaining ITs.^3^

A study conducted in the United Kingdom demonstrated that ITs and ACTs are considered the most demanding parts of the treatment. Professional guidance to patients who experienced a reduction in respiratory symptoms was to reduce routines but never to suspend them. Even with the increased flexibility and individualization of routines, the assisting team is often resistant to changes in treatment routines due to the fear that patients will not implement them again in case of an exacerbation.^2^

It is important to note that, in both studies, the included population had preserved lung function, not being representative of most of the Brazilian population with CF. These findings still hinder the extrapolation of these data to our referral centers in terms of reducing ITs and ACTs in the long term.^2^ ^,^ ^3^

Physical exercise has become increasingly important in the daily lives of patients, representing a significant area of intervention for physiotherapists and physical education professionals. With the reduction of ACT routines, the encouragement of routine exercise, both aerobic and anaerobic, is important for maintaining physical conditioning and preventing excessive weight gain, which may become a reality for patients using ETI.

For the first time, patients are optimistic about their future due to a reduction in symptoms and the possibility of reducing the burden imposed by treatment. It is up to the teams to customize the treatment carefully for each patient according to their new reality. The positive effects of reducing ITs and ACTs are associated with improvements in well-being, mental health, and social life. However, their effects on long-term lung function are yet to be described. It is necessary to act with caution and rigorously monitor routine changes until more evidence can guide future treatment guidelines for patients undergoing ETI.

## References

[1] Silva LVRFD, Athanazio RA, Tonon CR, Ferreira JC, Tanni SE (2024). Use of elexacaftor+tezacaftor+ivacaftor in individuals with cystic fibrosis and at least one F508del allele a systematic review and meta-analysis. J Bras Pneumol.

[2] Almulhem M, Harnett N, Graham S, Haq I, Visram S, Ward C (2022). Exploring the impact of elexacaftor-tezacaftor-ivacaftor treatment on opinions regarding airway clearance techniques and nebulisers TEMPO a qualitative study in children with cystic fibrosis, their families and healthcare professionals. BMJ Open Respir Res.

[3] Mayer-Hamblett N, Ratjen F, Russell R, Donaldson SH, Riekert KA, Sawicki GS (2023). Discontinuation versus continuation of hypertonic saline or dornase alfa in modulator treated people with cystic fibrosis (SIMPLIFY) results from two parallel, multicentre, open-label, randomised, controlled, non-inferiority trials. Lancet Respir Med.

