# Supplementary material for: Challenges in the treatment of cystic fibrosis in the era of CFTR modulators
Source: J Bras Pneumol. 2024 May 8;50(2):e20240107. doi: 10.36416/1806-3756/e20240107 (PMC11185157; doi:10.36416/1806-3756/e20240107)
Supplement: Related texts

# Authors’ reply

**Authors:** Luiz Vicente Ribeiro Ferreira da Silva, Rodrigo Abensur Athanazio, Carolina Rodrigues Tonon, Juliana Carvalho Ferreira, Suzana Erico Tanni

**Affiliations:** 1. Unidade de Pneumologia Pediátrica, Instituto da Criança e do Adolescente, Hospital das Clínicas, Faculdade de Medicina, Universidade de São Paulo, São Paulo (SP) Brasil.; 2. Divisão de Pneumologia, Instituto do Coração - InCor - Hospital das Clínicas, Faculdade de Medicina, Universidade de São Paulo, São Paulo (SP) Brasil.; 3. Departamento de Clínica Médica, Faculdade de Medicina de Botucatu, Botucatu, (SP) Brasil.

We received the correspondence entitled “Challenges in the treatment of cystic fibrosis in the era of CFTR modulators” and would like to thank the authors for bringing up such an important issue. As more evidence accumulates, CFTR modulators such as elexacaftor-tezacaftor-ivacaftor (ETI) stand for as a revolutionary therapeutic option for people with cystic fibrosis (CF). The magnitude of improvement in many key health outcomes of the disease is quite impressive^1^ and will certainly transform the scenario of CF care in the next few years.

We do agree with the authors of the correspondence that many uncertainties about standard CF care will emerge, including management of respiratory infections, use of mucolytics, and airway clearance therapies (ACTs). As stated in the correspondence, nebulized therapies and ACTs are indeed responsible for most of the treatment burden in CF, and towards that, the SIMPLIFY study^2^ aimed to investigate the safety of withdrawing hypertonic saline or dornase alfa use in people with CF treated with ETI. Although the study confirmed the non-inferiority outcome, it enrolled patients with well-maintained lung function and assessed the impact on lung function over a relatively brief period of six weeks.^2^. A recently published data from the SIMPLIFY substudy, however, focusing on individuals with CF and impaired lung function, demonstrated comparable results after discontinuation of hypertonic saline among those with FEV_1_ levels ranging from 40% to 69%.^3^ Consequently, we acknowledge that CF healthcare providers, while potentially making such decisions, should remain aware of associated risks and ensure vigilant and thorough monitoring of individuals when implementing these practices.

While we also believe that ACTs represent one of the main-stem aspects of CF care, a revision of the role of each professional composing the multidisciplinary team involved in CF care is anticipated. Doctors themselves should be observant to changes in life expectancy and possible complications related to aging. Psychologists should focus on behavior adjustments in the face of a new lifestyle and life expectancy. Physiotherapists may need to focus on muscular conditioning and rehabilitation of individuals who may have spent most of their lives with severe physical limitations.

Finally, we believe that we are witnessing a very exciting new era in CF. This is a much more optimistic scenario, although there is still plenty of uncertainties that must be adequately evaluated and investigated in the coming years in order to provide the best treatment options for individuals with CF.

## References

[1] Silva LVRFD, Athanazio RA, Tonon CR, Ferreira JC, Tanni SE (2024). Use of elexacaftor+tezacaftor+ivacaftor in individuals with cystic fibrosis and at least one F508del allele a systematic review and meta-analysis. J Bras Pneumol.

[2] Mayer-Hamblett N, Ratjen F, Russell R, Donaldson SH, Riekert KA, Sawicki GS (2023). Discontinuation versus continuation of hypertonic saline or dornase alfa in modulator treated people with cystic fibrosis (SIMPLIFY) results from two parallel, multicentre, open-label, randomised, controlled, non-inferiority trials. Lancet Respir Med.

[3] Nichols D, Gifford A, Russell R, Odem-Davis K, Young J, Amaro-Galvez R (2024). Assessing Safety of Discontinuing Hypertonic Saline in Those with Lower Forced Expiratory Volume in 1 Second after Elexacaftor/Tezacaftor/Ivacaftor. Ann Am Thorac Soc.
